# Serum protein α-klotho mediates the association between lead, mercury, and kidney function in middle-aged and elderly populations

**DOI:** 10.1265/ehpm.24-00296

**Published:** 2025-02-22

**Authors:** Lin Jiang, Tingting Guo, Xin Zhong, Yini Cai, Wanyu Yang, Jun Zhang

**Affiliations:** 1 Emergency Department, Beijing Ditan Hospital, Capital Medical University, Beijing, 100015 China; 2 Department of Nephrology, Zhujiang Hospital, Southern Medical University, Guangzhou Guangdong, 510280 China; 3 Department of Nephrology, Longgang Central Hospital of Shenzhen, Guangzhou Shenzhen, 518116 China

**Keywords:** Kidney function, α-Klotho, Lead, Mercury, Mediation, Estimated glomerular filtration

## Abstract

**Background:**

Heavy metals are significant risk factors for kidney function. Numerous studies have shown that exposure to heavy metals negatively correlates with kidney function through oxidative stress pathways, and serum α-klotho is linked to oxidative stress. However, the role of α-klotho in the relationship between blood lead, mercury, and kidney function remains unclear.

**Method:**

This study evaluated the mediating role of alpha-klotho in the relationship between lead, mercury and renal function, using data from the 2007–2016 National Health and Nutrition Examination Survey (NHANES) in U.S. adults aged 40–79. The sample included 11,032 participants, with blood lead, mercury, α-klotho, and other relevant covariates measured. Inductively coupled plasma mass spectrometry was used to assess blood lead and mercury levels, and enzyme-linked immunosorbent assay (ELISA) was employed to measure serum α-klotho. Kidney function was evaluated using estimated glomerular filtration rate (eGFR) based on creatinine levels. Multivariable linear regression was conducted to analyze the relationships between blood lead, mercury, α-klotho, and eGFR. A mediation analysis model was used to assess whether α-klotho influenced these associations.

**Results:**

We observed a significant association between blood lead and eGFR. Mediation analysis revealed that α-klotho accounted for 12.76% of the relationship between serum lead and eGFR in the NHANES population. Subgroup analysis showed that α-klotho mediated 12.43%, 6.87%, 21.50% and 5.44% of the relationship between blood lead and eGFR in women, middle-aged adults (40–59 years old), without cardiovascular disease and hypertension, respectively. However, α-klotho did not mediate the relationship between blood mercury and eGFR in terms of gender or age. This newly identified pathway may provide valuable insights for the prevention and treatment mechanisms related to kidney function impairment.

**Conclusion:**

We found that blood lead was associated with renal function. According to the results of subgroup analysis, for blood lead, serum α-klotho mediated the association in females, middle aged 60–79 years. The relationship between blood mercury and renal function was not clinically significant, and serum α-Klotho mediated the relationship between blood mercury and renal function without significant clinical significance.

## 1. Introduction

Chronic kidney disease (CKD) is an irreversible and progressive condition caused by multiple factors, with high morbidity and mortality rates. CKD can lead to kidney failure, cardiovascular complications, and premature death [1–3]. It is estimated that CKD affects 11–15% of the global population, and by 2040, it is expected to become the fifth leading cause of death worldwide, making it one of the fastest-growing causes of mortality [4]. Therefore, preventing CKD by reducing risk factors for kidney function decline is both crucial and necessary. With the rapid industrialization of the world, exposure to metals has increased. Since metals are easily absorbed into the bloodstream and affect various tissues, studying their impact on the kidneys is essential [5].

Heavy metals are widespread in the environment and can enter the human body through the skin, diet, and respiration, leading to neurological, metabolic, renal, cardiovascular, endocrine, and autoimmune diseases. Even at very low levels, they can cause functional damage to organs [6, 7]. Heavy metals such as lead and mercury are common pollutants associated with kidney disease [8]. In a prospective cohort study conducted in Sweden, which included 4,341 individuals aged 46–67 years over a long follow-up period, blood lead levels were found to be negatively associated with kidney function and positively associated with CKD incidence [9]. Navas-Acien confirmed that blood lead is an independent risk factor for decreased eGFR and proteinuria using data from the 1996–2006 NHANES database [10]. The role of blood mercury remains inconsistent across studies. In one NHANES study, lead exposure alone caused renal dysfunction in adults, whereas mercury exposure alone did not affect renal function [11]. However, another study involving 110 miners in Ghana found a link between blood mercury levels and reduced eGFR [12]. Oxidative stress is one of the key mechanisms through which lead and mercury toxicity damages kidney function [13–19].

The protein α-klotho is a member of the single-channel transmembrane protein family, which includes α-, β-, and γ-klotho isoforms. It is primarily expressed in the kidney, brain, and parathyroid glands [20, 21]. α-Klotho plays a crucial role in various physiological processes, including phosphate homeostasis, insulin signaling, oxidative stress regulation, vitamin D metabolism, and ion reabsorption. Notably, it contributes positively to the generation of antioxidant enzymes [22, 23]. Previous studies have shown that retinal pigment epithelial cells pretreated with α-klotho for 24 hours were protected from H_2_O_2_-induced cell death by enhancing the activity of antioxidants such as SOD2. In neurons treated with α-klotho, antioxidant stress analysis revealed a significant enhancement in the expression of thioredoxin/peroxiredoxin (Trx/Prx). Since Prx-2 is a key regulator of neuroprotection, α-klotho protects cells through antioxidant responses and the generation of antioxidant enzymes. Additionally, the transmembrane form of α-klotho acts as a co-receptor for fibroblast growth factor 23 (FGF-23), mediating FGF-23 activity in the kidney and regulating renal calcium and phosphorus metabolism [24]. Ischemic or toxic injury impairs oxidative phosphorylation in renal mitochondria. Recent studies have reported reduced α-klotho expression in renal tubular epithelial cells following the onset of acute kidney injury (AKI), leading to tubular epithelial damage and renal fibrosis [25]. Furthermore, α-klotho deficiency exacerbates injury after hypoxia or reoxygenation.

In summary, heavy metals such as lead and mercury impair renal function through various oxidative stress pathways, whereas α-klotho exhibits antioxidant properties. α-klotho may mediate the relationship between heavy metals (lead and mercury) and renal function was hypothesized. This potential association in a representative sample of US adults from NHANES was investigated. If lead and mercury influence renal function through α-klotho, this protein could serve as a preventive and therapeutic target for renal dysfunction associated with heavy metal exposure.

## 2. Methods

### 2.1. Study population

The Centers for Disease Control and Prevention (CDC) conducts the National Health and Nutrition Examination Survey (NHANES, available at: https://www.cdc.gov/nchs/nhanes/index.htm), a nationally representative survey of the noninstitutionalized U.S. civilian population. Laboratory tests and medical, dental, and physiological examinations are performed by trained medical personnel.

**Fig. 1:**
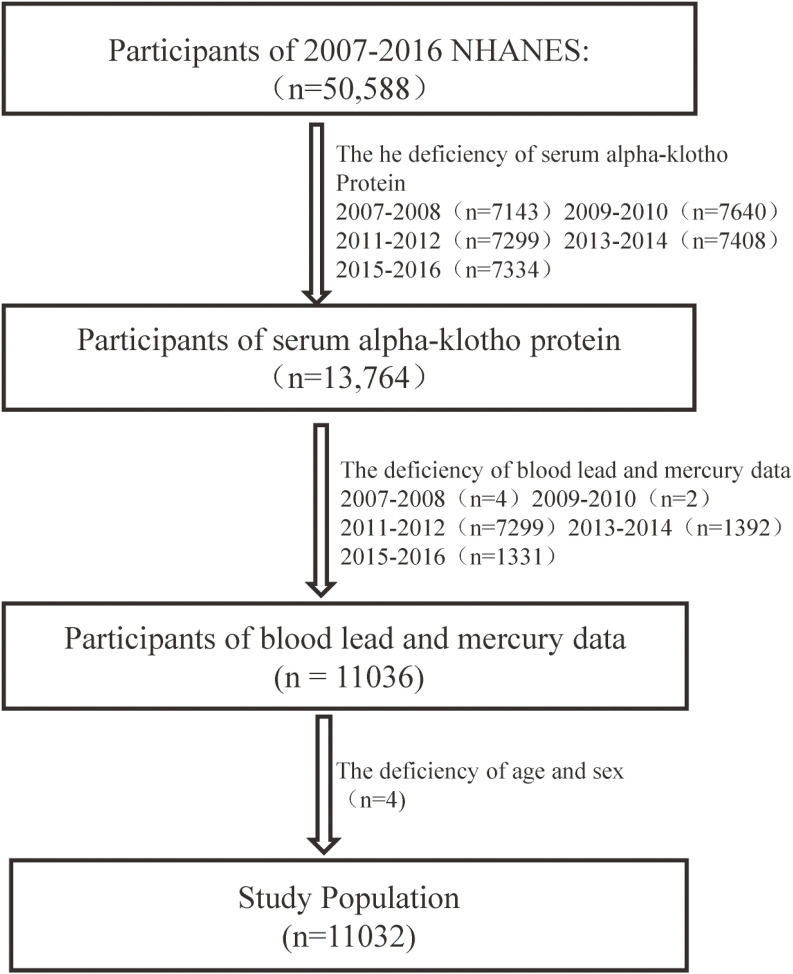
Flowchart for selecting analytical sample from NHANES

Our study analyzed NHANES data collected between 2007 and 2016, focusing on serum α-klotho protein levels. Since serum α-klotho measurements in the NHANES database were available only for participants aged 40–79, the data from five NHANES cycles (2007–2016) were combined, resulting in an initial sample of 50,588 voluntary participants [26]. Data on serum α-klotho levels were missing for 36,824 participants. Additionally, data were missing for blood lead and blood mercury levels in 2,728 participants, and sex and age information was unavailable for 4 participants. Consequently, the final study population included 11,032 participants (Fig. 1). The NHANES study protocol received approval from the Institutional Review Board of the National Center for Health Statistics (available at: https://www.cdc.gov/nchs/nhanes/irba98.htm). All participants provided both verbal and written consent for future studies (Fig. 1).

### 2.2 Determination of serum α-klotho levels

Serum α-klotho levels were analyzed in frozen serum samples from individuals aged 40 to 79, collected from the NHANES database between 2007 and 2016. Freshly frozen serum samples, stored at −80 °C, were shipped from the CDC to the Lipid Metabolism and Diabetes Research Laboratory, Division of Endocrinology and Nutrition, University of Washington, NW. Serum α-klotho levels were measured using an ELISA kit manufactured by IBL International, Inc. The average of two replicate analyses was used for quality assurance, and samples showing more than a 10% difference between replicates were reanalyzed. The assay sensitivity was 4.33 pg/mL. In 114 samples from healthy volunteers, the reference range was 285.8 to 1638.6 pg/mL, with a mean value of 698.0 pg/mL [27].

### 2.3. Determination of blood lead and blood mercury levels

Whole blood samples were collected at the Mobile Examination Center (MEC) and processed, stored, and transported according to the requirements of the National Center for Environmental Health (NCEH). All measurements were analyzed following the Laboratory Procedures Manual (LPM). Lead and mercury levels were determined using mass spectrometry after sample dilution. Inductively coupled plasma mass spectrometry (ICP-MS) measured blood lead and mercury concentrations. The lower limit of detection (LOD) was calculated for each element: 0.07 µg/dL for blood lead and 0.28 µg/L for blood mercury. For analytes below these LODs, values were estimated using the lowest LOD value divided by the square root of 2 (LOD/ √2 ), resulting in 0.05 µg/dL for blood lead and 0.14 µg/L for blood mercury. The accuracy and precision of all assays adhered to the quality control and quality assurance standards set by Environmental Laboratory Science [28].

### 2.4. Function assessment

Renal function assessment in this study was primarily performed using two parameters: estimated glomerular filtration rate (eGFR) and the urinary albumin-to-creatinine ratio (UACR). The main focus was on eGFR, which was calculated using the 2021 CKD Epidemiology Collaboration (CKD-EPI) equation [29], based on serum creatinine levels. UACR served as the outcome measure for sensitivity analysis and was calculated using the formula [30]: UACR (mg/g) = urinary albumin level (mg/dL)/urinary creatinine level (g/dL). Both urinary albumin and creatinine levels were obtained from urine samples.

### 2.5. Covariates

The covariate data included sex, age, education, race, annual household income, body mass index (BMI), physical activity, and comorbidities such as hypertension, diabetes, and atherosclerotic heart disease. Age was categorized into two groups: 40–59 years and 60–79 years. BMI was calculated by dividing weight in kilograms by the square of height in meters, with categories defined as follows: normal (18.5–25), overweight (25–30), and obese (>30). Race was categorized into non-Hispanic white, non-Hispanic black, Mexican American, and other Hispanic. Annual household income was grouped into three categories: less than $20,000, $20,000–75,000, and greater than $75,000. Educational attainment was classified as less than high school, high school, and more than high school. Physical activity was categorized as high-intensity, moderate-intensity, and low-intensity. Comorbidities included diabetes, hypertension, and cardiovascular disease, with cardiovascular disease encompassing a history of coronary heart disease, angina, stroke, or heart attack.

### 2.6. Data analysis

Since serum α-klotho protein, blood lead, blood mercury, and eGFR data were skewed, the variables were log-transformed to reduce skewness. Categorical variables were assessed by calculating percentages, while continuous variables were assessed by determining the mean (±SD). Nonparametric continuous variables were analyzed using the interquartile range (IQR). Pearson’s correlation coefficients were calculated for eGFR, blood lead, blood mercury, and α-klotho levels, including their log-transformed values.

Linear regression analysis was performed to evaluate the correlation between renal function (dependent variable) and blood lead and blood mercury (independent variables). Beta coefficients and 95% confidence intervals (CIs) were calculated. Similarly, linear regression analysis was used to evaluate the correlation between serum α-klotho (dependent variable) and blood lead and blood mercury (independent variables), with beta coefficients and 95% CIs computed. Models were adjusted for age, gender, race, education, income, BMI, physical activity, hypertension, diabetes, and cardiovascular disease.

Additionally, the potential mediating role of α-klotho in the association between blood lead, blood mercury, and renal function was explored. The bootstrapping method was used to calculate mediating effects and estimate 95% CIs. Log-transformed values of metals (lead, mercury) were used as exposure variables, log-transformed serum α-klotho as the mediator, and log-transformed eGFR as the outcome variable. The following outcomes were assessed: the effect of metals on α-klotho; the effect of α-klotho on renal function; the direct effect of metals on renal function; the overall effect of metals on renal function considering α-klotho protein, with the proportion of the mediating factor calculated using the formula: (βtotal effect − βdirect effect)/βtotal effect × 100%.

Subgroup analyses were conducted to explore whether age, sex, cardiovascular disease, and hypertension moderated the mediating role of serum α-klotho between metals and eGFR. To handle different variable types (continuous, dichotomous, unordered categorical, and ordered categorical), multiple imputations were performed using MICE for covariate data with less than 5% missing values. Linear regression was applied for continuous variables, and binary logistic regression was used for categorical variables, following Rubin’s rules. The results from the five datasets were pooled to create a single dataset with combined effects, minimizing errors [31, 32]. To verify the robustness of the results post-imputation, sensitivity analyses were performed to examine the role of serum α-klotho in the relationship between metals and UACR.

Weighted estimates were applied according to NHANES guidelines, using MEC weights. All statistical analyses were conducted using R (version 4.3.1) and SPSS Statistics (version 27), with a two-sided p-value of <0.05 considered statistically significant. This study adhered to the guidelines for cross-sectional studies to enhance observational reporting in epidemiology.

## 3. Results

### 3.1. Characteristics of participants

A total of 11,032 eligible participants aged 40–79 years from 2007 to 2016 were selected from the NHANES database (Table 1). The mean age (standard deviation) of the participants was 57.76 (10.89) years. The blood lead levels (median [IQR]) and blood mercury levels (median [IQR]) in the total study population were 0.07 [0.05, 0.10] µmol/L and 0.93 [0.50, 1.90] µg/L, respectively. The α-klotho level in the overall study population was 802.80 [654.65, 996.20] pg/mL (Table 1).

**Table 1:** Characterization of serum α-klotho levels according to study participants (n = 11032).

	**N (%)**	**Median and IQR of serum α-klotho (in pg/mL)**		**p-value**
Age (year)				<0.001
40–59	5974 (54%)	821.70	(671.93, 1,022.70)	
60–79	5058 (46%)	780.80	(635.05, 967.05)	
Gender				<0.001
Female	5,624 (51%)	822.85	(664.30, 1,030.83)	
Male	5,408 (49%)	783.60	(644.88, 966.25)	
Ethnicity				<0.001
Non-Hispanic blacks	2,194 (20%)	829.95	(649.10, 1,085.05)	
Black Mexican-American	1,733 (16%)	799.70	(647.20, 985.00)	
Non-Hispanic whites	4,862 (44%)	788.75	(649.43, 962.98)	
Others	2,243 (20%)	825.50	(677.20, 1,004.65)	
educational attainment (%)				0.003
University degree and above	5,375 (49%)	812.20	(663.05, 1,011.70)	
high school education	4,098 (37%)	796.70	(643.10, 982.15)	
Less than high school education	1,552 (14%)	792.55	(649.95, 985.98)	
Unclear	4 (<0.1%)	723.85	(616.33, 824.00)	
decline to answer	3 (<0.1%)	669.50	(516.15, 755.25)	
Annual household income (%)				0.7
<20000	4,198 (38%)	805.60	(658.13, 998.60)	
20000–75000	5,485 (50%)	801.20	(651.20, 996.30)	
>75000,	958 (8.7%)	795.10	(654.60, 972.98)	
Unclear	155 (1.4%)	811.20	(669.70, 1,027.40)	
decline to answer	236 (2.1%)	815.90	(671.78, 1,033.55)	
BMI (%)				0.007
Normal	2,592 (23%)	816.95	(664.48, 1,019.10)	
Overweight	3,903 (35%)	798.20	(653.30, 986.05)	
Obese	4,537 (41%)	798.60	(650.20, 991.50)	
eGFR				<0.001
≥90	5300	826.85	(680.03,1022.73),	
<90	5732	774.90	(628.80, 969.38)	
Physical activity (%)				0.6
Low level sports	3,326 (30%)	802.20	(643.00, 1,003.48)	
Moderate intensity sports	4,579 (42%)	802.40	(658.75, 996.35)	
High-intensity exercise	3,104 (28%)	804.95	(659.88, 986.23)	
Hypertension (%)				<0.001
Yes	5,118 (46%)	792.70	(635.53, 988.80)	
No	5,901 (53%)	813.70	(669.50, 1,002.10)	
Unclear	13 (0.1%)	760.10	(559.70, 881.20)	
diabetes (%)				0.11
Yes	1,941 (18%)	790.30	(631.00, 1,008.90)	
No	9081 (82.8%)	805.20	(658.30, 993.60)	
Unclear	10 (<0.1%)	730.60	(652.28, 867.95)	
cardiovascular disease (%)				<0.001
Yes	1,462 (13%)	767.70	(616.00, 950.48)	
No	9570 (87%)	809.45	(659.73, 1,002.50)	
year (%)				<0.001
2007–2008	3,002 (27%)	803.75	(653.10, 988.18)	
2009–2010	2,896 (26%)	792.10	(644.30, 992.63)	
2011–2012	2,453 (22%)	842.40	(682.50, 1,039.40)	
2013–2014	1,375 (12%)	806.80	(672.45, 984.20)	
2015–2016	1,306 (12%)	751.95	(618.85, 952.23)	

### 3.2 Median serum klotho by quartiles of lead and mercury

**Fig. 2:**
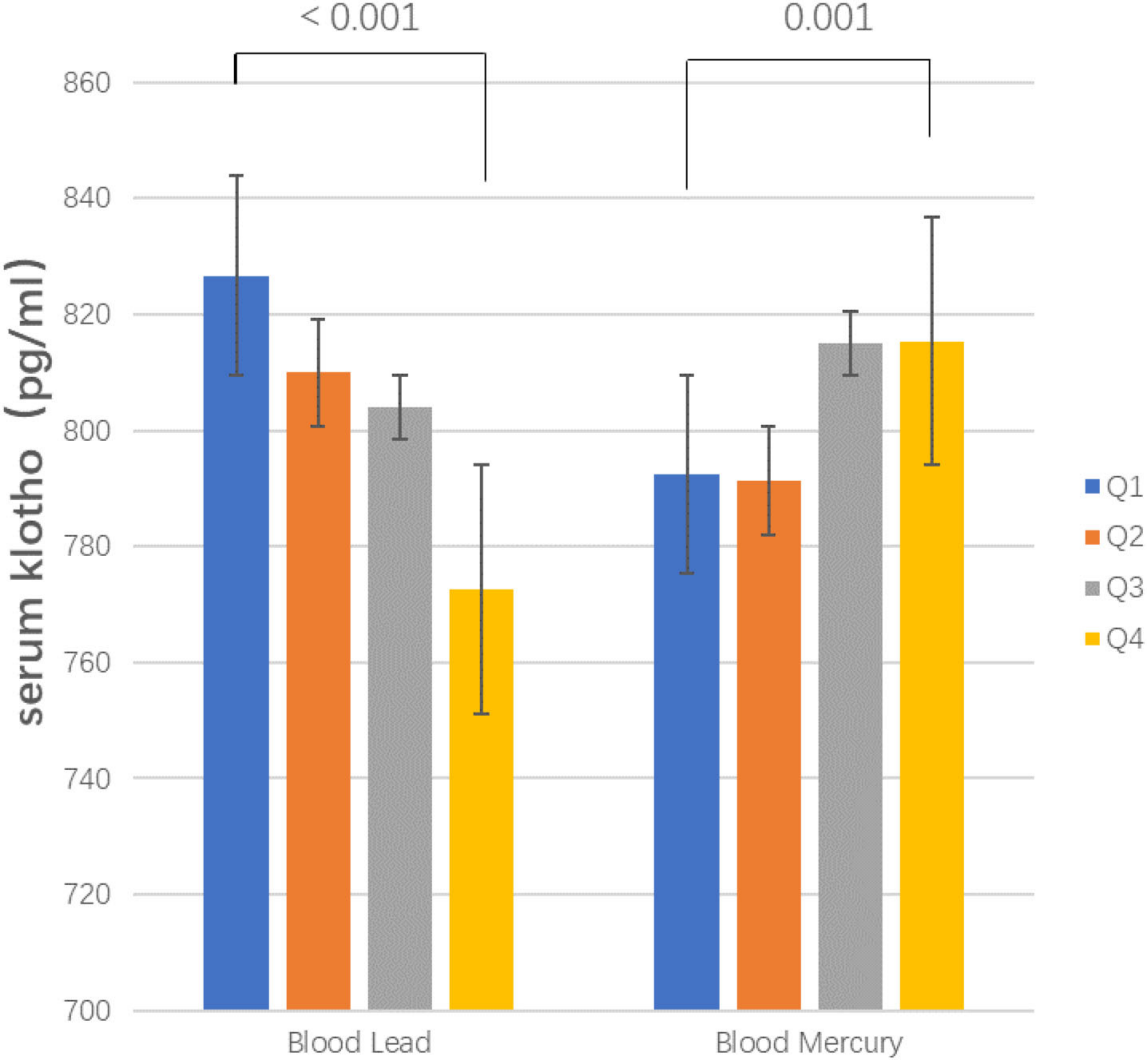
Median klotho levels for quartiles of blood lead and blood mercury at baseline

Figure 2 shows the median serum klotho levels for the quartiles of lead and mercury. The median serum klotho levels demonstrated a statistically significant decreasing trend with increasing blood lead levels (median (IQR)) pg/ml: Q1: 826.70 (Cl 95%: 672.85, 1032.95), Q2: 809.95 (Cl 95%: 658.00, 1006.95), Q3: 804.05 (Cl 95%: 655.38, 979.50), Q4: 772.60 (Cl 95%: 628.90, 965.10; p < 0.001). In contrast, blood mercury quartiles showed a statistically significant upward trend (median (IQR) pg/ml: Q1: 792.40 (Cl 95%: 639.45, 823.04), Q2: 791.35 (Cl 95%: 647.20, 986.13), Q3: 815.00 (Cl 95%: 659.15, 1006.50), Q4: 815.30 (Cl 95%: 669.43, 1006.43; p = 0.001)).

### 3.3. Relationship between serum α-klotho, blood lead, blood mercury, and eGFR

Table 2 shows the log-transformed matrix of relationships among α-klotho, blood lead, blood mercury, and eGFR. Serum α-klotho exhibited a negative correlation with blood lead (−0.076, p < 0.001), a positive correlation with blood mercury (0.045, p < 0.05), a negative correlation with blood lead (−0.187, p < 0.001), a positive correlation with blood mercury (0.33, p < 0.001), and a positive correlation between serum α-klotho and eGFR (0.119, p < 0.001).

**Table 2:** Pearson’s correlation coefficients among log-transformed lead, mercury, eGFR, and α-klotho levels

	**Klotho**		**Blood lead**		**Blood mercury**		**eGFR**		**Log klotho**		**Log blood lead**		**Log blood mercury**	

	**(pg/mL)**		**(umol/L)**		**(umol/L)**		**(ml/min/1.73m2)**		**(pg/mL)**		**(umol/L)**		**(umol/L)**	
	**r**	**p-value**	**r**	**p-value**	**R**	**p-value**	**r**	**p-value**	**r**	**p-value**	**r**	**p-value**	**r**	**p-value**
Klotho	1													
Blood lead	−0.056	<0.001	1											
Blood mercury	0.041	<0.001	0.05	<0.001	1									
eGFR	0.119	<0.001	−0.127	<0.001	0.04	<0.001	1							
Log Klotho	0.957	<0.001	−0.064	<0.001	0.004	<0.001	0.139	<0.001	1					
Log blood lead	−0.069	<0.001	0.823	<0.001	0.071	<0.001	−0.184	<0.001	−0.076	<0.001	1			
Log blood mercury	0.039	<0.001	0.045	<0.001	0.732	<0.001	0.029	<0.001	0.045	<0.001	0.078	<0.001	1	
Log eGFR	0.122	<0.001	−0.0149	<0.001	0.041	<0.001	0.946	<0.001	0.149	<0.001	−0.187	<0.001	0.33	<0.001

### 3.4. Linear relationship between blood mercury and eGFR, UACR

Table 3 shows the log-transformed model of the relationship between eGFR and blood mercury. A 1% increase in blood mercury was associated with a 0.8% increase in eGFR after adjusting for all relevant variables (β = 0.0086; 95% CI: 0.027, 0.0145; p < 0.001). To further analyze the results, the association between log-transformed UACR using sensitivity analyses was examined to confirm the findings. A positive correlation between blood mercury and UACR was found; however, when adjusted for Model III, the results were not statistically significant (Table 3).

**Table 3:** The relationship among log-transformed lead, mercury, eGFR, and α-klotho levels

**Total study population** **weighted β coefficient (95% CI) for log (eGFR)**			
	Model1	Model2	Model3
log(Lead) (umol/L)	−0.0200 (−0.0324, −0.0075)***	−0.0230 (−0.0353, −0.0106)***	−0.0269 (−0.0400, −0.0138)***
log(Mercury) (umol/L)	0.0116 (0.0055, 0.0176)***	0.0107 (0.0047, 0.0168)***	0.0086 (0.027, 0.0145)***

	Model1	Model2	Model3
log(Lead) (umol/L)	0.0419 (−0.0048, 0.0887)	0.0264 (−0.0194, 0.0723)	0.0470 (0.0063, 0.0876)*
log(Mercury) (umol/L)	−0.0619 (−0.0880, −0.0359)***	−0.0507 (−0.0769, −0.0244)***	−0.0253 (−0.0509, 0.0001)

	Model1	Model2	Model3
log(Lead) (umol/L)	−0.0335 (−0.0485, −0.0184)***	−0.0327 (−0.0480, −0.0175)***	−0.0413 (−0.0565, −0.0261)***
log(Mercury) (umol/L)	0.0150 (0.0044, 0.0255)**	0.0132 (0.0028, 0.0235)*	0.0119 (0.0012, 0.0226)*

Model I. Adjusted for age, gender, race and sex; (*p < 0.05, **p < 0.01, ***p < 0.001)

Model II: Adjusted for age, gender, race, and sex, education, and income;

Model III: Adjusted for age, gender, Race, education, Income, BMI, physical activity, hypertension, diabetes and cardiovascular diseases.

### 3.5 Linear relationship between serum α-klotho protein and blood lead and blood mercury

Table 3 shows the log-transformed model of the relationship between serum α-klotho protein, blood lead, and blood mercury. After adjusting for all relevant variables, a 1% increase in blood lead was associated with a 4.13% decrease in serum α-klotho protein (β = −0.0413; 95% CI: −0.0565, −0.0261). Blood mercury showed a mild positive correlation with serum α-klotho protein after full adjustment, with a 1.19% increase in serum α-klotho protein for each 1% increase in blood mercury (β = 0.0119; 95% CI: 0.0012, 0.0226; Table 3).

### 3.6. Mediating role of serum α-klotho in blood lead and mercury

A mediation analysis was conducted to determine whether α-klotho mediated the relationship between log-transformed metals (blood lead, blood mercury) and renal function, adjusting for potential confounders. The results indicated a significant negative indirect effect of blood lead on eGFR via serum α-klotho (β = −0.0203; 95% CI: −0.0342, −0.01; p < 0.001), suggesting a partial mediating role of serum α-klotho. After controlling for α-klotho, the direct effect of blood lead remained significant (β = −0.0177; 95% CI: −0.0309, −0.01; p < 0.001), indicating that blood lead exerts both direct and indirect effects. The indirect effect of blood lead on eGFR mediated by serum α-klotho was approximately 12.76% (8.00, 23). In contrast, blood mercury showed a positive indirect effect (β = 0.0006; 95% CI: 0.0003, 0; p < 0.05) and a positive direct effect (β = 0.0091; 95% CI: 0.0040, 0.01; p < 0.001) on eGFR via serum α-klotho, with an effect of approximately 6.10% (2.56, 29) mediated by serum α-klotho (Table 4). To further investigate the mediating role of serum α-klotho, a sensitivity analysis was conducted using UACR. The results showed that serum α-klotho did not mediate the relationship between blood lead, blood mercury, and UACR (Table 4).

**Table 4:** Mediating role of log-transformed α-klotho in the association between standardized log-transformed lead and mercury and eGFR

	**Total study population**			
	**weighted β coefficient (95% CI) for eGFR**			
Medicator α-klotho	total effect	indirect effect	direct effect	proportion Mediated, %(95%)
log(Lead) (umol/L)	−0.0203 (−0.0324, −0.01)***	−0.0026 (−0.0042, 0)***	−0.0177 (−0.0309, −0.01)***	12.76 (8.00, 23)***
log(Mercury) (umol/L)	0.091 (0.0040, 0.01)***	0.0006 (0.0003, 0)*	0.0085 (0.0028, 0.01)**	6.10 (2.56, 29)*
	weighted β coefficient (95% CI) for UACR			
Medicator α-klotho	total effect	indirect effect	direct effect	proportion Mediated, %(95%)
log(Lead) (umol/L)	−1.8701 (−1.9764, −1.47)***	‘0.0160 (−0.0076, 0.03)	−1.8861 (−1.9793, −1.48)***	-
log(Mercury) (umol/L)	−0.2035 (−0.1963, 0.11)	−0.0036 (−0.0085, 0)	−0.2000 (−0.1929, 0.12)	-
stratification by age				
weighted β coefficient (95% CI) for eGFR				
Age	total effect	indirect effect	direct effect	proportion Mediated, %(95%)
Pb				
40–59	−0.0386 (−0.048, −0.02)***	−0.0027 (−0.0036, 0)	−0.0360 (−0.0448, −0.02)***	6.87 (4.14, 11)***
60–79	−0.0644 (−0.0908, −0.05)***	−0.0013 (−0.0063, 0)*	−0.0631 (−0.0854, −0.04)***	2.03 (1.45, 10)*
Hg				
40–59	5.38e-03 (−2.38e-03, 0.01)	7.54e-04 (−5.57e-05, 0)	4.63e-03 (−2.74e-03, 0.01)	-
60–79	0.0059 (−0.0004, 0.02)	0.0019 (0.0002, 0)*	0.0040 (−0.0024, 0.02)	-
weighted β coefficient (95% CI) for UACR				
Age	total effect	indirect effect	direct effect	proportion Mediated, %(95%)
Pb				
40–59	−1.9142 (−1.9881, −1.37)***	0.0182 (−0.0144, 0.03)	−1.9323 (−1.999, −1.39)***	-
60–79	−1.3308 (−1.7143, −1.01)***	0.0021 (−0.0043, 0.03)	−1.3329 (−1.7294, −1.02)***	-
Hg				
40–59	−0.2250 (−0.3008, 0.05)	−0.0052 (−0.0075, 0)	−0.2199 (−0.3014, 0.05)	-
60–79	0.1619 (−0.0783, 0.35)	−0.0030 (−0.0193, 0)	0.1649 (−0.0676, 0.35)	-
stratification by gender				
weighted β coefficient (95% CI) for eGFR				
Gender	total effect	indirect effect	direct effect	proportion Mediated, %(95%)
Pb				
male	−0.02360 (−0.0404, −0.01)***	−0.0027 (−0.0055, 0)***	−0.0209 (−0.0362, −0.01)***	11.56 (7.54, 31)***
female	−0.0264 (−0.0462, −0.01)***	−0.0033 (−0.0039, 0)**	−0.0231 (−0.0442, −0.01)**	12.43 (2.04, 17)**
Hg				
male	−0.0002 (−0.0067, 0.01)	0.0016 (0.0002, 0)*	−0.0020 (−0.0078, 0)	-
female	0.0154 (0.0101, 0.02)***	0.0010 (−0.0004, 0)	0.0144 (0.0096, 0.02)***	-
weighted β coefficient (95% CI) for UACR				
Gender	total effect	indirect effect	direct effect	proportion Mediated, %(95%)
Pb				
male	−1.9587 (−1.8996, −1.20)***	0.0297 (−0.0148, 0.05)	−1.9883 (−1.9195, −1.21)***	-
female	−2.1227 (−2.2969, −1.58)***	−0.0037 (−0.0125, 0.02)	−2.1190 (−2.3072, −1.59)***	-
Hg				
male	−0.3345 (−0.3330, 0.06)	−0.0183 (−0.0180, 0.01)	−0.3162 (−0.3285, 0.07)	-
female	−0.0592 (−0.1166, 50.30)	0.0012 (−0.00921, 0)	−0.0604 (−0.1162, 0.30)	-

stratification by Cardiovascular_disease				
weighted β coefficient (95% CI) for eGFR				
Cardiovascular_disease	total effect	indirect effect	direct effect	proportion Mediated, %(95%)
Pb				
Yes	−0.0719 (−0.1503, −0.05)***	−0.0074 (−0.0096, 0)***	−0.0645 (−0.1461, −0.05)***	10.29 (4.07, 12)***
No	−0.0129 (−0.0283, −0.01)***	−0.0028 (−0.0036, 0)***	−0.0101 (−0.0257, −0.01)**	21.50 (6.69,30)***
Hg				
Yes	0.0375 (0.0011, 0.04)	0.0025 (−0.0019, 0)*	−0.034987 (0.0005, 0.04)*	-
No	0.0041 (0.0020, 0.01)**	0.0004 (0.0002, 0)**	0.0037 (0.0012, 0.01)*	9.26 (3.02,45)*
weighted β coefficient (95% CI) for UACR				
Cardiovascular_disease	total effect	indirect effect	direct effect	proportion Mediated, %(95%)
Pb				
Yes	0.0074 (0.0583, 0.33)**	0.0146 (−0.0031, 0.02)	−0.0072 (0.0542, 0.32)**	-
No	0.0775 (0.0435, 0.12)***	0.0013 (−0.0007, 0.02)	0.0762 (0.0410, 0.12)***	-
Hg				
Yes	−0.0707 (−0.1376, 0.03)	−0.0048 (−0.01, 0)	−0.0659 (−0.1350, 0.03)	-
No	−0.02445 (−0.0458, 0.01)	−0.0002 (−0.0021, 0)	−0.0243 (−0.0453, 0.01)	-

stratification by Hypertension				
weighted β coefficient (95% CI) for eGFR				
Hypertension	total effect	indirect effect	direct effect	proportion Mediated, %(95%)
Pb				
Yes	−0.0521 (−0.0761, −0.03)***	−0.0028 (−0.0065, 0)**	−0.0493 (−0.0718, −0.03)***	5.44 (2,4, 14)**
No	−0.0067 (−0.0224, 0)	−0.0009 (−0.0024, 0)***	−0.0058 (−0.0208, 0)	
Hg				
Yes	1.11e-02 (9.58e-04, 0.02)*	1.54e-03 (−4.69e-04, 0)	9.60e-03 (−1.21e-05, 0.02)*	-
No	0.0082 (0.0019, 0.01)**	0.0004 (0.0001, 0)**	0.0078 (0.0014, 0.01)*	4.73 (1.73,33)**
weighted β coefficient (95% CI) for UACR				
Hypertension	total effect	indirect effect	direct effect	proportion Mediated, %(95%)
Pb				
Yes	0.1670 (0.1008, 0.25)***	0.0077 (0.0007, 0.01)*	0.1593 (0.0946, 0.24)***	4.6 (0.5,9)*
No	−0.0200 (−0.0135, 0.08)	−0.0025 (−0.0052, 0)	−0.0175 (−0.0116, 0.08)	-
Hg				
Yes	−0.0269 (−0.0514, 0.04)	−0.0030 (−0.0053, 0)	−0.0239 (−0.0507, 0.04)	-
No	−0.03350 (−0.0644, −0.01)***	0.0011 (−0.0004, 0)	−0.0346 (−0.0659, −0.01)***	-

Adjusted models for age, sex, race, education, income, BMI, hypertension, diabetes, cardiovascular disease, *p < 0.05, **p < 0.01, ***p < 0.001

### 3.7 Subgroup analysis

To examine the adjustment of age, sex, cardiovascular disease and hypertension for the mediating role of serum α-klotho in the association between blood lead, mercury, and eGFR, we performed subgroup analyses. After fully adjusting for covariates, we found that serum α-klotho protein mediated the relationship between blood lead and eGFR under those stratification, with more pronounced mediation for 40–59 years, hypertension, without cardiovascular_disease women. The results showed that indirect effect value of α-klotho on the association between middle-aged adults, females, hypertension, without cardiovascular_disease were (95% CI) = −0.0027 ((−0.0055, 0), p < 0.001) was mediated by 6.87% (4.14, 11), (95% CI) = −0.0033 ((−0.0039,0), p < 0.1), with a mediation proportion of 12.43% (2.04,17), (95% CI) = −0.00284 ((−0.00648, 0), p < 0.01), with a mediation proportion of 5.44% (2.4, 14), and β (95% CI) = −0.0028 ((−0.0036, 0), p < 0.001), with a mediation proportion of 21.50% (6.69, 30), respectively.

In contrast, serum α-klotho protein did not mediate the relationship between blood mercury and renal function in the mediation analysis after fully adjusted modeling for blood mercury. After sensitivity analysis with UACR instead of eGFR, the results showed that serum α-klotho did not mediate the relationship between blood lead, blood mercury, and UACR (Table 4).

## 4. Discussion

This study selected 11,032 participants from the NHANES database (2007–2016), aged 40–79 years, to explore the mediating role of serum α-klotho in the relationship between blood lead, mercury, and renal function (eGFR). After adjusting for covariates such as age, gender, family income, hypertension, and coronary atherosclerotic heart disease, our study found a significant negative linear correlation between blood lead and eGFR, as well as a positive linear correlation between blood mercury and eGFR. Additionally, the mediation analysis revealed that serum α-klotho mediated this association to some extent. The mediating role of serum α-klotho in the relationship between blood lead and eGFR was more significant than that between blood mercury and eGFR. In the subgroup analysis, α-klotho did not mediate the relationship between blood mercury and eGFR. In contrast, for blood lead, especially in women, middle-aged individuals, those with hypertension, and those without cardiovascular disease, α-klotho played a more pronounced mediating role in the relationship between blood lead and eGFR. These findings provide insights into the oxidative stress pathways linking blood lead, mercury, and renal function.

The relationship between lead, mercury, and kidney function has been extensively studied. Imbalances in the oxidative and antioxidant systems are significantly linked to decreased kidney function. Some studies have shown that lead induces excessive production of reactive oxygen species (ROS), depleting endogenous ROS scavengers and disrupting various transport proteins, which leads to kidney damage [33–35]. Lead can also disrupt the balance of heme oxygenase activity, causing damage or apoptosis of renal tubular epithelial cells, impairing the reabsorption function of the renal tubules, and ultimately leading to decreased kidney function and the development of kidney diseases [36].

Accumulation of mercury ions in proximal tubular cells can lead to oxidative stress, as mercury acts as a potent oxidant. It has been demonstrated that exposure of rats to organic forms of mercury induces increased synthesis of glutathione (GSH) to counteract the oxidative stress caused by exposure to Hg^2+^ [37]. A cross-sectional study in Gansu, China, also found that kidney function impairment was positively correlated with heavy metals, and 8-OHG might play an important mediating role (2.6%–86.9%). Additionally, mercury can directly alter mitochondrial oxidation and participate in various protein oxidation reactions, such as binding to metallothionein in the cytoplasm of renal tubular epithelial cells, which causes a decrease in γ-glutamyl transpeptidase [38].

The present study found that after adjusting for covariates, blood lead levels were negatively correlated with renal function levels and positively correlated with proteinuria. These findings are consistent with previous studies, which further validate the deleterious effects of blood lead concentrations on renal function. In contrast, the effect of blood mercury on renal function in the present study is not consistent with previous studies, suggesting that a true protective effect of mercury is unlikely. Instead, a healthy lifestyle (fish consumption) may have confounded the results by exerting a protective effect through fish-derived long-chain omega-3 polyunsaturated fatty acids, which could explain the absence of harmful effects of mercury in this low-exposure population [39].

In the general population, the main sources of mercury exposure are elemental mercury from dental amalgams and methylmercury ingested through the consumption of fish. In both cases, exposure is relatively low and may not be sufficient to cause kidney damage. If a healthy lifestyle has an effect on mercury exposure, then the results of studies on lead must also be considered. Some studies have shown that the amount of lead in red blood cells is related to fish consumption. However, in our study, the association between kidney disease and lead was unlikely to be confounded by a healthy lifestyle, as the risk estimate for lead would have decreased rather than increased. The study found that the risk estimates for lead gradually increased [40]. Thus, the association between blood lead and renal function remains more relevant.

Overall, our findings emphasize the importance of the effects of blood lead and mercury on renal function. However, the mechanisms behind this association are not fully understood. In summary, blood lead may affect renal function through oxidative stress pathways, and renal impairment is also associated with serum α-klotho, which is involved in the oxidative stress pathway. Therefore, these findings suggest that blood lead may affect renal function through the serum α-klotho protein. Thus, the results of this study suggest that blood lead can affect renal function through the overproduction of ROS, and blood lead is negatively correlated with serum α-klotho. α-Klotho can affect renal function through oxidative stress. A recent study suggested that the level of α-klotho protein decreases, and the reduction in α-klotho impairs renal mitochondrial fatty acid oxidation, leading to renal tubular injury and fibrosis [25]. Therefore, this relationship may be explained by the following mechanism: blood lead and mercury may affect renal function by reducing α-klotho, which in turn disrupts renal mitochondrial metabolic pathways, ultimately resulting in kidney damage. Further studies are needed to confirm the mechanism behind this association.

## 5. Strengths and limitations

To the best of our knowledge, this is the first study to report the relationship between serum α-klotho levels and renal function, mediated by blood lead and mercury levels, in a representative sample of US adults. It contributes to a new understanding of how blood lead and mercury mediate renal function through serum α-klotho. Additionally, this study utilized a large sample, with all data collected through standardized interviews, physical examinations, and laboratory tests, minimizing potential sources of measurement bias. However, this study also has several limitations. First, it was observed that serum α-klotho mediated the negative relationship between blood lead and eGFR, as well as UACR. However, unlike previous studies, blood mercury was negatively correlated with eGFR but did not correlate with UACR. Further research with larger sample sizes and diverse populations is needed to elucidate the relationship and mechanisms between blood mercury and renal function. Second, although adjusted for confounders to the best of our ability, some unobserved confounders may still exist. It is not feasible to rule out factors such as dietary influences, genetic variations, or exposure to other pollutants. Third, this study is cross-sectional in nature, which limits our ability to infer causal relationships between lead, mercury, and kidney function. Therefore, longitudinal observational studies are needed to fully understand the relationship between blood lead, mercury, and renal function, as well as the potential biological role of serum α-klotho.

## 6. Conclusion

This study highlights the important role of serum α-klotho in the relationship between blood lead, mercury, and renal function, further emphasizing the effects of these heavy metals on kidney health. It suggests that oxidative stress pathways may be involved in this relationship. Specifically, our findings regarding the role of serum α-klotho in the mechanism linking lead and mercury exposure to renal function may inform strategies for preventing and treating renal impairment. This could open new avenues for the treatment and prevention of renal diseases. If preventive measures are implemented, such as drinking purified water and avoiding areas contaminated with heavy metals, the risk of exposure could be reduced. Additionally, greater attention should be paid to the protection of individuals in occupations with high exposure to heavy metals. In cases where avoidance or mitigation of damage is not possible, improving the body’s antioxidant capacity may help prevent kidney damage, for example, through the administration of antioxidants. Furthermore, our findings underscore the importance of improving living environments and controlling heavy metal pollution, which should be prioritized by health researchers and government authorities.

## Declarations

### Availability of data and materials

The datasets used and/or analyzed during the current study are available from the corresponding author on reasonable request.

## References

[1] Levey AS, Coresh J. Chronic kidney disease. Lancet. 2012;379(9811):165–80. 21840587 10.1016/S0140-6736(11)60178-5

[2] Hu MC, Shi M, Gillings N, Flores B, Takahashi M, Kuro-O M, . Recombinant α-Klotho may be prophylactic and therapeutic for acute to chronic kidney disease progression and uremic cardiomyopathy. Kidney Int. 2017;91(5):1104–14. 28131398 10.1016/j.kint.2016.10.034 PMC5592833

[3] Jager KJ, Kovesdy C, Langham R, Rosenberg M, Jha V, Zoccali C. A single number for advocacy and communication-worldwide more than 850 million individuals have kidney diseases. Kidney Int. 2019;96(5):1048–50. 31582227 10.1016/j.kint.2019.07.012

[4] Foreman KJ, Marquez N, Dolgert A, Fukutaki K, Fullman N, McGaughey M, . Forecasting life expectancy, years of life lost, and all-cause and cause-specific mortality for 250 causes of death: reference and alternative scenarios for 2016–40 for 195 countries and territories. Lancet. 2018;392(10159):2052–90. 30340847 10.1016/S0140-6736(18)31694-5 PMC6227505

[5] Ekong EB, Jaar BG, Weaver VM. Lead-related nephrotoxicity: a review of the epidemiologic evidence. Kidney Int. 2006;70(12):2074–84. 17063179 10.1038/sj.ki.5001809

[6] Jannetto PJ, Cowl CT. Elementary Overview of Heavy Metals. Clin Chem. 2023;69(4):336–49. 36945128 10.1093/clinchem/hvad022

[7] Wu W, Zhang K, Jiang S, Liu D, Zhou H, Zhong R, . Association of co-exposure to heavy metals with renal function in a hypertensive population. Environ Int. 2018;112:198–206. 29275245 10.1016/j.envint.2017.12.023

[8] Yuan TH, Jhuang MJ, Yeh YP, Chen YH, Lu S, Chan CC. Relationship between renal function and metal exposure of residents living near the No. 6 Naphtha Cracking Complex: A cross-sectional study. J Formos Med Assoc. 2021;120(10):1845–54. 33933337 10.1016/j.jfma.2021.04.009

[9] Afrifa J, Essien-Baidoo S, Ephraim RKD, Nkrumah D, Dankyira DO. Reduced egfr, elevated urine protein and low level of personal protective equipment compliance among artisanal small scale gold miners at Bibiani-Ghana: a cross-sectional study. BMC Public Health. 2017;17(1):601. 28655297 10.1186/s12889-017-4517-z PMC5488392

[10] Wennberg M, Bergdahl IA, Hallmans G, Norberg M, Lundh T, Skerfving S, . Fish consumption and myocardial infarction: a second prospective biomarker study from northern Sweden. Am J Clin Nutr. 2011;93(1):27–36. 21048056 10.3945/ajcn.2010.29408

[11] Sommar JN, Svensson MK, Björ BM, Elmståhl SI, Hallmans G, Lundh T, . End-stage renal disease and low level exposure to lead, cadmium and mercury; a population-based, prospective nested case-referent study in Sweden. Environ Health. 2013;12:9. 23343055 10.1186/1476-069X-12-9 PMC3566948

[12] Cai R, Zheng YF, Bu JG, Zhang YY, Fu SL, Wang XG, . Effects of blood lead and cadmium levels on homocysteine level in plasma. Eur Rev Med Pharmacol Sci. 2017;21(1):162–6. 28121341

[13] Rahman Z, Singh VP. The relative impact of toxic heavy metals (THMs) (arsenic (As), cadmium (Cd), chromium (Cr)(VI), mercury (Hg), and lead (Pb)) on the total environment: an overview. Environ Monit Assess. 2019;191(7):419. 31177337 10.1007/s10661-019-7528-7

[14] Tian X, Shan X, Ma L, Zhang C, Wang M, Zheng J, . Mixed heavy metals exposure affects the renal function mediated by 8-OHG: A cross-sectional study in rural residents of China. Environ Pollut. 2023;317:120727. 36427825 10.1016/j.envpol.2022.120727

[15] Huang Y, Wan Z, Zhang M, Hu L, Song L, Wang Y, . The association between urinary metals/metalloids and chronic kidney disease among general adults in Wuhan, China. Sci Rep. 2023;13(1):15321. 37714886 10.1038/s41598-023-42282-z PMC10504376

[16] Moubarz G, Mohammed AMF, Saleh IA, Shahy EM, Helmy MA. Nephrotoxic effect of heavy metals and the role of DNA repair gene among secondary aluminum smelter workers. Environ Sci Pollut Res Int. 2023;30(11):29814–23. 36418822 10.1007/s11356-022-24270-4 PMC9995418

[17] Mishra M, Nichols L, Dave AA, Pittman EH, Cheek JP, Caroland AJV, . Molecular Mechanisms of Cellular Injury and Role of Toxic Heavy Metals in Chronic Kidney Disease. Int J Mol Sci. 2022;23(19). 10.3390/ijms231911105 PMC9569673 36232403

[18] Su F, Zeeshan M, Xiong LH, Lv JY, Wu Y, Tang XJ, . Co-exposure to perfluoroalkyl acids and heavy metals mixtures associated with impaired kidney function in adults: A community-based population study in China. Sci Total Environ. 2022;839:156299. 35643130 10.1016/j.scitotenv.2022.156299

[19] Liang JH, Pu YQ, Liu ML, Bao WW, Zhang YS, Hu LX, . Synergistic impact of co-exposures to whole blood metals on chronic kidney disease in general US adults: a cross-sectional study of the National Health and Nutrition Examination Survey 2011–2020. Environ Sci Pollut Res Int. 2023;30(53):113948–61. 37858011 10.1007/s11356-023-30177-5

[20] Kuro-O M. The Klotho proteins in health and disease. Nat Rev Nephrol. 2019;15(1):27–44. 30455427 10.1038/s41581-018-0078-3

[21] Biyik I, Ozatik FY, Albayrak M, Ozatik O, Teksen Y, Ari NS, . The effects of recombinant klotho in cisplatin-induced ovarian failure in mice. J Obstet Gynaecol Res. 2021;47(5):1817–24. 33611838 10.1111/jog.14700

[22] Edmonston D, Grabner A, Wolf M. FGF23 and klotho at the intersection of kidney and cardiovascular disease. Nat Rev Cardiol. 2024;21(1):11–24. 37443358 10.1038/s41569-023-00903-0

[23] Wang Y, Ran L, Lan Q, Liao W, Wang L, Wang Y, . Imbalanced lipid homeostasis caused by membrane αKlotho deficiency contributes to the acute kidney injury to chronic kidney disease transition. Kidney Int. 2023;104(5):956–74. 37673285 10.1016/j.kint.2023.08.016

[24] Kim D, Lee S, Choi JY, Lee J, Lee HJ, Min JY, . Association of α-klotho and lead and cadmium: A cross-sectional study. Sci Total Environ. 2022;843:156938. 35753483 10.1016/j.scitotenv.2022.156938

[25] He H, Chen X, Miao D, Zhang H, Wang Y, He X, . Composite Dietary Antioxidant Index and Plasma Levels of Soluble Klotho: Insights from NHANES. Oxid Med Cell Longev. 2023;2023:3524611. 36798687 10.1155/2023/3524611 PMC9928515

[26] Inker LA, Eneanya ND, Coresh J, Tighiouart H, Wang D, Sang Y, . New Creatinine- and Cystatin C-Based Equations to Estimate GFR without Race. N Engl J Med. 2021;385(19):1737–49. 34554658 10.1056/NEJMoa2102953 PMC8822996

[27] Cai Q, Hu S, Qi C, Yin J, Xu S, Hou FF, . Serum Anti-Aging Protein α-Klotho Mediates the Association between Diet Quality and Kidney Function. Nutrients. 2023;15(12). 10.3390/nu15122744 PMC10301566 37375648

[28] Mera-Gaona M, Neumann U, Vargas-Canas R, López DM. Evaluating the impact of multivariate imputation by MICE in feature selection. PLoS One. 2021;16(7):e0254720. 34320016 10.1371/journal.pone.0254720 PMC8318311

[29] White IR, Royston P, Wood AM. Multiple imputation using chained equations: Issues and guidance for practice. Stat Med. 2011;30(4):377–99. 21225900 10.1002/sim.4067

[30] Harari F, Sallsten G, Christensson A, Petkovic M, Hedblad B, Forsgard N, . Blood Lead Levels and Decreased Kidney Function in a Population-Based Cohort. Am J Kidney Dis. 2018;72(3):381–9. 29699886 10.1053/j.ajkd.2018.02.358

[31] Navas-Acien A, Tellez-Plaza M, Guallar E, Muntner P, Silbergeld E, Jaar B, . Blood cadmium and lead and chronic kidney disease in US adults: a joint analysis. Am J Epidemiol. 2009;170(9):1156–64. 19700501 10.1093/aje/kwp248 PMC2781739

[32] Jain RB. Co-exposures to toxic metals cadmium, lead, and mercury and their impact on unhealthy kidney function. Environ Sci Pollut Res Int. 2019;26(29):30112–8. 31420836 10.1007/s11356-019-06182-y

[33] Chung S, Chung JH, Kim SJ, Koh ES, Yoon HE, Park CW, . Blood lead and cadmium levels and renal function in Korean adults. Clin Exp Nephrol. 2014;18(5):726–34. 24276216 10.1007/s10157-013-0913-6

[34] Wang L, Li J, Li J, Liu Z. Effects of lead and/or cadmium on the oxidative damage of rat kidney cortex mitochondria. Biol Trace Elem Res. 2010;137(1):69–78. 19902158 10.1007/s12011-009-8560-1

[35] Lv X, Ren M, Xu T, Gao M, Liu H, Lin H. Selenium alleviates lead-induced CIK cells pyroptosis and inflammation through IRAK1/TAK1/IKK pathway. Fish Shellfish Immunol. 2023;142:109101. 37758100 10.1016/j.fsi.2023.109101

[36] Woods JS, Ellis ME. Up-regulation of glutathione synthesis in rat kidney by methyl mercury. Relationship to mercury-induced oxidative stress. Biochem Pharmacol. 1995;50(10):1719–24. 7503776 10.1016/0006-2952(95)02075-6

[37] Agrawal S, Flora G, Bhatnagar P, Flora SJS. Comparative oxidative stress, metallothionein induction and organ toxicity following chronic exposure to arsenic, lead and mercury in rats. Cell Mol Biol (Noisy-le-grand). 2014;60(2):13–21. 24970117

[38] Bridges CC, Zalups RK. The aging kidney and the nephrotoxic effects of mercury. J Toxicol Environ Health B Crit Rev. 2017;20(2):55–80. 28339347 10.1080/10937404.2016.1243501 PMC6088787

[39] Ebrahimi M, Khalili N, Razi S, Keshavarz-Fathi M, Khalili N, Rezaei N. Effects of lead and cadmium on the immune system and cancer progression. J Environ Health Sci Eng. 2020;18(1):335–43. 32399244 10.1007/s40201-020-00455-2 PMC7203386

[40] Aaseth J, Alexander J, Alehagen U, Tinkov A, Skalny A, Larsson A, . The Aging Kidney-As Influenced by Heavy Metal Exposure and Selenium Supplementation. Biomolecules. 2021;11(8). 10.3390/biom11081078 PMC8391790 34439746

